# Sesame‐enriched delights: A comparative exploration of physicochemical and sensory attributes in fine and whole wheat flour cookies

**DOI:** 10.1002/fsn3.4343

**Published:** 2024-08-11

**Authors:** Sittara Noori Naqvi, Muhammad Liaquat, Abeer Kazmi, Shella Sammi, Amir Ali, Juan Pedro Luna‐Arias, Izzat Ullah Sherzad

**Affiliations:** ^1^ Food Science and Technology University of Haripur Haripur Pakistan; ^2^ The State Key Laboratory of Freshwater Ecology and Biotechnology, the Key Laboratory of Aquatic Biodiversity and Conservation of Chinese Academy of Sciences, Institute of Hydrobiology Chinese Academy of Sciences Wuhan Hubei China; ^3^ University of Chinese Academy of Sciences Beijing China; ^4^ Nanoscience and Nanotechnology Program Center for Research and Advanced Studies of the National Polytechnic Institute Mexico City Mexico; ^5^ Department of Horticulture, Faculty of Agriculture Nangarhar University Jalalabad Nangarhar Afghanistan

**Keywords:** cookies, fortification, NARC‐2011, physicochemical, sesame, wheat

## Abstract

Cookies are an exceptional energy source due to their elevated fat and carbohydrate content. Beyond their delectable taste, they are also rich in essential nutrients, including valuable proteins and minerals. This study evaluated the potential of the wheat variety NARC‐2011 for cookie production, focusing on nutritional enhancement by adding white sesame seeds at different proportions (5%, 10%, and 15%) to both whole and fine wheat flour. White sesame seeds were added to cookies mainly for their visual appeal, creating a nice contrast with the dough. They also have a mild flavor that complements the cookie without overwhelming it. Besides, they pack essential nutrients like protein, fiber, calcium, and iron, making the cookies more nutritious. The physical, chemical, and sensory properties of the different cookies were evaluated using standard methods. In terms of physical parameters, fine wheat flour cookies exhibited a diameter (46.45–50.47 mm), thickness (8.47–9.77 mm), and spreading factor (5.16–5.46 mm), and whole wheat flour cookies exhibited a diameter (48.47–52.31 mm), thickness (9.22–10.73 mm), and spreading factor (4.87–5.25 mm). Chemical analysis revealed moisture (5.78%–7.66%), fat (10.89%–16.16%), fiber (6.10%–8.46%), ash (4.82%–7.40%), protein (0.74%–1.40%), non‐fiber carbohydrates (63.67%–67.55%) for fine wheat flour cookies, and moisture (5.67%–7.39%), fat (10.89%–16.16%), fiber (11.47%–15.98%), ash (0.54%–0.83%), protein (5.65%–8.13%), non‐fiber carbohydrates (57.86%–66.55%), total phenolic content (2.86 mg/g), flavonoids (1.46 mg/g), and antioxidant activity (80.76%) in whole wheat flour cookies with sesame fortification. Gas chromatography revealed higher unsaturated fatty acids (83.22%) in NARC‐2011 wheat oil compared to white sesame seed oil (79.78%). In sensory evaluations, cookies fortified with 10% sesame seeds in fine wheat flour received the highest level of acceptability from the panelists. On the other hand, cookies made from whole wheat flour fortified with 15% sesame seeds garnered the maximum acceptability ratings from the panelists. In conclusion, supplementing NARC‐2011 wheat flour with sesame seeds, whether in fine or whole wheat form, improves the quality of cookies and nutritional content while offering appropriate sensory attributes at particular sesame seed levels.

## Graphical abstract

This study explored the nutritional enhancement of cookies by incorporating white sesame seeds (10%, 20%, and 30%) into NARC‐2011 wheat flour. Chemical analysis revealed varied parameters in fine and whole wheat flour cookies, with increased phenolic content, flavonoids, and antioxidant activity in sesame‐fortified cookies. Sensory evaluations favored cookies with 20% sesame seeds in fine wheat flour and 30% in whole wheat flour, highlighting the potential of NARC‐2011 wheat flour supplemented with sesame seeds to improve cookie quality and nutritional content.

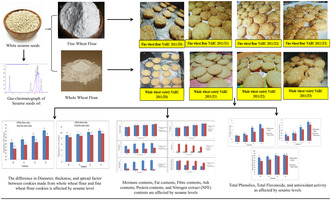

## 1 INTRODUCTION

Due to their wonderful sweetness, biscuits have earned worldwide appeal among youngsters. The use of fortified composite flour in typical baked goods such as cookies and biscuits is a new invention in the baking business. These products may be used in large‐scale feeding programs and relief efforts in the aftermath of natural disasters because of their improved nutritional quality and other convenience characteristics like simple packaging, extended shelf life, and ready‐to‐eat composition (Pratima & Yadav, 2000). Due to their high fat and carbohydrate content, cookies are an excellent source of energy. In addition to being tasty, they contain critical nutrients such as protein and minerals (Kure et al., 1998). Food fortification may be a possible solution to nutritional deficiencies in developing nations. The objective of food fortification is to increase the consumption of a certain vitamin or nutrients deemed to be deficient in the food supply (Sharma et al., 2024).

Sesame seeds (*Sesamum indicum*) are small, oval, flat, and have a nutty flavor. It is often believed to have originated in the tropical areas of Africa (RMRDC, 2004). Sesame has significant amounts of protein (18%–23.5%), carbs (13%), and oil (44%–52.5%) (Bamigboye et al., 2010; Kahyaoglu & Kaya, 2006). Raw, dehydrated, roasted, or sugar‐coated seeds are all appropriate routes of intake. Additionally, it is utilized as a paste in several traditional regional soups (Chemonics, 2002). The nutritional value of sesame seeds may be improved by preconsumption preparation (such as soaking, roasting, and germination). Its seeds are extensively employed in a variety of global culinary traditions (Mohamed et al., 2007). Sesame seeds are an excellent source of superior protein. Not only does it include zinc, a necessary mineral for the metabolism of carbohydrates, lipids, and proteins, but it also contains iron, one of the most concentrated forms of iron available. Therefore, individuals of all ages may profit from it (Gopalan et al., 1989).

Wheat (*Triticum aestivum*), a Poaceae (Gramineae) family member, is a highly esteemed cereal crop. It provides more calories and protein to the global diet than other cereal crops, which is why over 33 percent of the global population depends on it as their primary source of nutrition (maize, rice, barley, oats, rye, and sorghum). Despite being eaten at a lower rate than maize, wheat was anticipated to have the second‐highest global production in 2022/23 at 803.1 million tons, behind only maize (FAO, 2023).

The nutritional value of cereal, particularly wheat‐based goods, is increasingly recognized as a crucial weapon in the fight against diet‐related illnesses such as obesity, cardiovascular disease, and type 2 diabetes. Accumulating data shows that the health advantages of a diet rich in whole‐grain cereals are most attributable to the increased consumption of specific minerals, phytochemicals, and dietary fiber (Fardet, 2010).

Wholegrain foods also include bioactive substances that may help prevent chronic illness, such as phytochemicals like phytoestrogens, phenols, antioxidants, and fermentable carbohydrates like dietary fiber, resistant starch, or oligosaccharides (Adom et al., 2005; Baublis et al., 2000; Jones, 2006; Hirawan et al., 2010; Sethi et al., 2023). Although these advantages are evident, not enough wholegrain products are ingested daily (O'Neil et al., 2010). Manufacturers have thus developed novel product lines, such as breakfast cereals with enhanced fiber from whole grains (Franz & Sampson, 2006; Zainuddin et al., 2014).

Wholegrain wheat is rich in fiber, which enhances digestive health and influences cardiovascular health. Dietary fiber promotes weight loss and provides a feeling of fullness. Eating whole grains increases metabolism, limits fat absorption, and lowers blood levels of harmful cholesterol (Ghattas et al., 2008; Marquart et al., 2006). Consumption of whole grains has been connected with a decreased risk of cardiovascular disease and numerous malignancies, as well as better blood lipid and glucose levels, less insulin resistance, and increased fiber and other nutritional intakes (Franz & Sampson, 2006; Madsen et al., 2024; McKeown et al., 2002).

Due to their high fat and carbohydrate content, cookies are an excellent source of energy. In addition to being tasty, they contain critical nutrients such as protein and minerals (Kure et al., 1998). Food fortification may be a possible solution to nutritional deficiencies in developing nations. The objective of food fortification is to increase the consumption of a certain vitamin or nutrients deemed to be deficient in the food supply (Sharma et al., 2024).

Several writers have reported that, in addition to being antioxidants, sesame extracts also exhibit antibacterial and antimutagenic characteristics, making them beneficial in several food‐preservation and nutraceutical applications (Reshma et al., 2010). Atherosclerosis, low‐density lipoprotein oxidation, cancer, platelet aggregation, and other cardiovascular disorders may be slowed down by consuming sesame seeds and sesame seed oil (Saleem et al., 2011).

The main aims of this research work were to determine the suitability of wheat variety NARC‐2011, physiochemical characteristics of sesame‐fortified cookies made from fine and whole wheat flour, the sensory and organoleptic properties of fortified cookies during storage, and the best‐fortified cookies for consumers in terms of nutrition and sensory characteristics.

## 2 MATERIALS AND METHODS

The study was conducted at the University of Haripur, Department of Food Science and Technology, and Nuclear Institute for Food and Agriculture, Peshawar. Wheat (NARC‐2011) and white sesame seeds were purchased from NARC‐Islamabad. Citric acid, Hydrochloric acid (HCl), Sulfuric acid (H_2_SO_4_), Sodium hydroxide (NaOH), Ammonia solution (NH_4_OH), Aluminum chloride (ALCL_3_), Sodium carbonate (Na_2_CO_3_), Ethanol (C_2_H_5_OH), Methanol (CH_3_OH), Gallic acid (C_7_H_6_O_5_), Sodium methoxide (CH_3_NaO), and Hexane (C_6_H_14_) were purchased from Sigma Aldrich and Fisher Scientific, USA.

### 2.1 Cookies preparation

The method of Qayyum et al. (2017) was adopted with a slight modification. In 200 g of wheat flour, 5%, 10%, and 15% sesame seeds were mixed for fortification in cookie preparation by using a laboratory‐scale planetary mixer (LabMaster LMPM‐10) with a mixing duration of 5 min at medium speed (approximately 200 rpm). Egg yolk (20 g), milk (10 mL), 1 drop of vanilla essence, 1 g baking powder, and sesame seeds were added to it, and then all the ingredients were mixed until the texture of the dough became smooth and plain. The dough was kept for 20 min, and then the biscuits were shaped and placed in the oven (Model: PEL Electric oven EO‐53) for 25 min at 180°C. After being removed from the pans, the cookies were cooled to room temperature, packed in airtight polythene bags, and stored at room temperature for 6 days. For the control experiment, cookies were made without sesame seeds in addition to both fine and whole wheat flour cookies (Table 1).

**TABLE 1:** Cookies formulation (whole/fine wheat cookies).

Treatments in whole/fine wheat flour	Sesame seeds (white) in whole/fine wheat flour	Wheat flour in whole/fine wheat flour
T0	0 g	200 g
T1	10 g	190 g
T2	20 g	180 g
T3	30 g	170 g

## 3 PROXIMATE ANALYSIS OF COOKIES

### 3.1 Moisture content

Moisture content was evaluated following the method of Handa et al. (2012). China dishes were properly dried in the hot air oven at 150°C. Then 10 g of each sample was taken in a china dish and heated in the hot air oven (Memmert UN110 Plus) preheated to 150°C for about 16 h. Dried samples were put in desiccators until they reached a constant weight.

Moisture content was obtained using the following equation:

$$\text{Moisture content}\;\left(\%\right)=\frac{W1-W2}{W1}\times 100$$

W1 = weight of sample before drying, W2 = weight of sample after drying.

### 3.2 Ash content

The AOAC (2000) No. 940.26 method was pursued to determine the ash content. In preweighed crucibles, a 2 g sample (moisture‐free) was taken and placed in a muffle furnace (Carbolite Gero CWF 1100) for 5 h at 550°C, till a grayish‐white residue was obtained. The crucibles were then shifted to desiccators to cool them. Ash content was calculated by using the following formula:

$$\mathrm{Ash}\;\text{content}\;\left(\%\right)=\frac{W2-W1}{\mathrm{Ws}}\times 100$$

where W2 = Weight of crucible with ash, W1 = Weight of crucible without ash, Ws = Weight of sample.

### 3.3 Crude fat

The AOAC method no. 983.23 (1990) was followed for the determination of fat content. Soxhlet apparatus was employed for this purpose. In a thimble, 5 g of sample (dried) was taken, and the mouth of the thimble was covered with cotton wool. A preweighed dried empty receiving flask was attached to the Soxhlet apparatus to complete the extraction assembly. Hexane was used as a solvent. After attaching the condenser to the apparatus, the flask was heated on the hot plate for about 4 h with a regular flow of water through the condenser. The solvent was evaporated, and the weight of the flask containing oil was calculated. The crude fat percentage was calculated using the following formula:

$$\text{Crude}\;\mathrm{fat}\;\left(\%\right)=\frac{W2-W1}{\mathrm{Ws}}\times 100$$

where W2 = Weight before extraction, W1 = Weight after extraction, Ws = Weight of sample.

### 3.4 Crude protein

The Micro‐Kjeldhal technique was used for the determination of the crude protein content. In a Kjeldhal flask, 1.5 g of dried powdered sample was combined with 1.5 g of digestion mixture, including 20 g of K_2_SO_4_ and 20 g of copper sulfate (CuSO_4_·5H_2_O), along with 1 mL of water and 7 mL of concentrated H_2_SO_4_. The combination underwent digestion for close to an hour at a temperature of 200°C. Following digestion, the flask was chilled, and the mixture was diluted with distilled water to a level of 50 mL in a volumetric flask. The aliquot of 5 mL was then moved to the distillation apparatus, and 5 mL of a solution containing 50% sodium hydroxide was added to it. After distilling the mixture, ammonia was collected in boric acid with a concentration of 4% and a few drops of an indicator (0.5 g of bromocresol green and 0.1 g of methyl red per 100 mL of ethanol at a pH of 4.5). Titration with 0.01 N hydrochloric acid (HCL) was used to assess the amount of ammonium hydroxide (NH_4_OH) present in the sample. In addition to this, a blank titration was performed. The formula used to compute the percentage of nitrogen is as follows.

$$\%\;\text{Nitrogen}=\frac{\left(S-B\right)\times N\;\text{of}\;\text{acid}\times 0.014\times \text{dilution}}{\text{Weight}\;\text{of}\;\text{sample}}\times 100$$

where S = Sample titration reading, B = Blank titration reading, N = Normality of acid.

The percent protein was determined by multiplying the nitrogen content with the factor 6.25 (Tribold & Aurand, 1963).

### 3.5 Crude fiber

The determination of crude fiber was done by Handa et al. (2012), using method No. 991.43. A fat‐free and dried sample (5 g) was poured into a beaker and digested by heating with a H_2_SO_4_ solution (1.25%) for 30 min. The mixture was filtered after heating and washed thoroughly with distilled water. This was followed by heating with NaOH (1.25%) solutions again for 30 min. The obtained residue was then filtered and washed with distilled water. The residue was transferred to the preweighted crucibles and ignited in a muffle furnace (Carbolite Gero CWF 1100) at 550°C (2–3 h) till white ash formed.

% crude fiber was determined using the following formula:

$$\%\;\text{crude}\;\text{fibre}=\frac{\mathrm{Wi}-\mathrm{Wf}}{\mathrm{Wi}}\times 100$$

where W = weight, Wi = initial weight, Wf = final weight.

### 3.6 Total carbohydrates

The nitrogen‐free extract (NFE), which represents carbohydrates, was calculated by subtracting the sum of moisture, ash, crude fat, crude protein, and crude fiber percentages from 100.

## 4 PHYTOCHEMICAL ANALYSIS

### 4.1 Sample preparation

The sample of 1 g was placed in 20 mL of 80% methanol, and then the mixture was centrifuged (Eppendorf 5424 Centrifuge) at 3000 rpm for 10 min. The supernatant was decanted, and the residue was again dissolved in 10 mL of methanol (80%) and centrifuged at 3000 rpm for 5 min, and then both supernatants were combined. This aliquot was used for the determination of total phenolic contents (TPC) and DPPH radical scavenging activity.

### 4.2 Determination of total phenolic content (TPC)

The diluted extracts of 1 mL were oxidized with 2.5 mL of Folin‐Ciocalteaus reagent (10%), followed by neutralization with 2 mL of sodium carbonate (7.5%). The mixture was kept in the dark (for 45 min), and the absorbance was measured at 765 nm using a spectrophotometer (UV‐9200, Beijing Beifen‐Ruili Analytical Instrument Co. Ltd., China). The standard used in this assay was gallic acid (Santos et al., 2014).

### 4.3 Determination of total flavonoids (TFC)

In a flask, a 0.5 mL sample was withdrawn. Subsequently, 0.1 mL of AlCl_3_ solution (10%), 1.5 mL of methanol, and 0.1 mL of potassium acetate were added. This mixture was then supplemented with 2.8 mL of distilled water. The sample was incubated for 30 min, followed by measuring the optical density at 415 nm.

### 4.4 Calculation

The measured absorbance value was utilized in the standard curve equation, Y = aX + b, where X represents the concentration of flavonoids in mg/g, a and b are constants specific to the calibration curve, and Y is the absorbance value. Subsequently, the calculated concentration of flavonoids (X) was inserted into the formula Flavonoids mg/g = (X × dilution)/(Weight of sample × 1000), where dilution refers to the dilution factor applied during sample preparation.

### 4.5 DPPH radical scavenging activity

The stable radical (DPPH) was used for the assessment of the free radical scavenger activity of the extracts (Berardini et al., 2005). DPPH extracts were added to 0.2 mM of DPPH in methanol. The mixture was left for 30 min at room temperature, and absorbance was taken at 517 nm. After 30 min at room temperature, the absorbance was recorded at 517 nm. Percent scavenging activity was determined as the ratio of the absorption of the sample reactive to the control (0.1 mM DPPH solutions without extract). DPPH activity was expressed as the inhibition percentage and calculated using the following formula:

$$\text{Radical}\;\text{scavenging}\;\text{activity}\;\left(\%\right)=\frac{\mathrm{Ac}-\mathrm{As}}{\mathrm{Ac}}\times 100$$

where As = Absorbance of sample, Ac = Absorbance of control.

### 4.6 Fatty acid determination

Gas chromatography (Agilent 7890A) was used to analyze the sesame seed and wheat oil to measure the levels of fatty acids. The technique that was used was developed by Hougen and Bodo (1973). With the assistance of soxhlet apparatus and rotatory equipment, oil was extracted from each of the samples (sesame seed and whole wheat grain). In a test tube measuring 10 mL by 75 mL, 1 mL of methylating solution and half mL of petroleum ether were poured. In each of the test tubes, a small amount of oil was dripped in from each of the samples. The loop was cleaned with petroleum ether at every step. Following the completion of the mixing process, the tube was sealed with a trainer top and left to rest at room temperature for 30 min. After adding one ml of distilled water or 1 mL of sodium chloride, the mixture was stirred and left to stand for 10 min. In gas chromatography equipment that had a glass column that was 2.1 m long and 3 mm in diameter on the inside, 1 mL of oil from the top layer was injected. A flame ionization detector was used. The temperature in the injector was 210°C, while that in the column was 230°C. At a flow rate of 50 mL per minute, hydrogen gas was employed as the carrier. The fatty acids were identified by comparing the retention periods of the fatty acid methyl esters standards to those of the fatty acid. A computer integrator was used for the immediate recording of the percent concentration of each fatty acid.

### 4.7 Sensory evaluation of cookies

The sensory evaluation of cookies packed in polythene bags was conducted for 30 days at room temperature. Cookies prepared with sesame seeds were noted with various numbers and presented to 3 panels for sensory evaluation at the Department of Food Science and Technology. They were informed to rate sensory attributes using control group cookies. The evaluation of cookies was based on the following attributes: color, taste, aroma, texture, and overall acceptability/quality on a hedonic scale having 9 points as described by Peryam and Pilgrim (1957).

### 4.8 Physical analysis

The following procedures from AACC (2000) were used to ascertain the diameter (width), thickness, and spread factor of whole and fine wheat cookies.

### 4.9 Diameter

Six cookies were laid out with their edges touching to measure the diameter (*D*). Using a ruler, we were able to determine the overall diameter of all six cookies in millimeters. To ensure accurate readings, the cookies were turned at an angle of 90°C. This was done one more time, and the results were reported in millimeters for the average diameter.

### 4.10 Thickness

Six cookies were stacked on top of one another to get an accurate reading of the thickness (*T*). Using a ruler, the overall height was measured in millimeters to provide an accurate reading. This procedure was carried out 3 times to get an average value, and the findings were expressed in millimeters. For the creation of round biscuits, the primary tool of choice is the biscuit cutter. This shaper is available in a variety of sizes and enables bakers to precisely cut out uniform circles of dough, ensuring even baking and aesthetic consistency (Mushtaq et al., 2010).

### 4.11 Spread factor

By using the following formula in combination with the diameter and thickness measurements, the spread factor (SF) was calculated:

$$\mathrm{SF}=\frac{D}{T}\times \mathrm{CF}\times 10$$

where CF is a correction factor at constant atmospheric pressure. Its value was 1.0 in this case.

### 4.12 Statistical analysis

Results were subjected to statistical analysis by using a two‐way analysis of variance (ANOVA) as required. Statistical differences with a *p*‐value under .05 were considered significant, and a comparison of mean values was carried out by applying the LSD method using Statistix 8.1 software (Steel & Torrie, 1980).

## 5 RESULTS AND DISCUSSION

### 5.1 Moisture content

**FIGURE 1:**
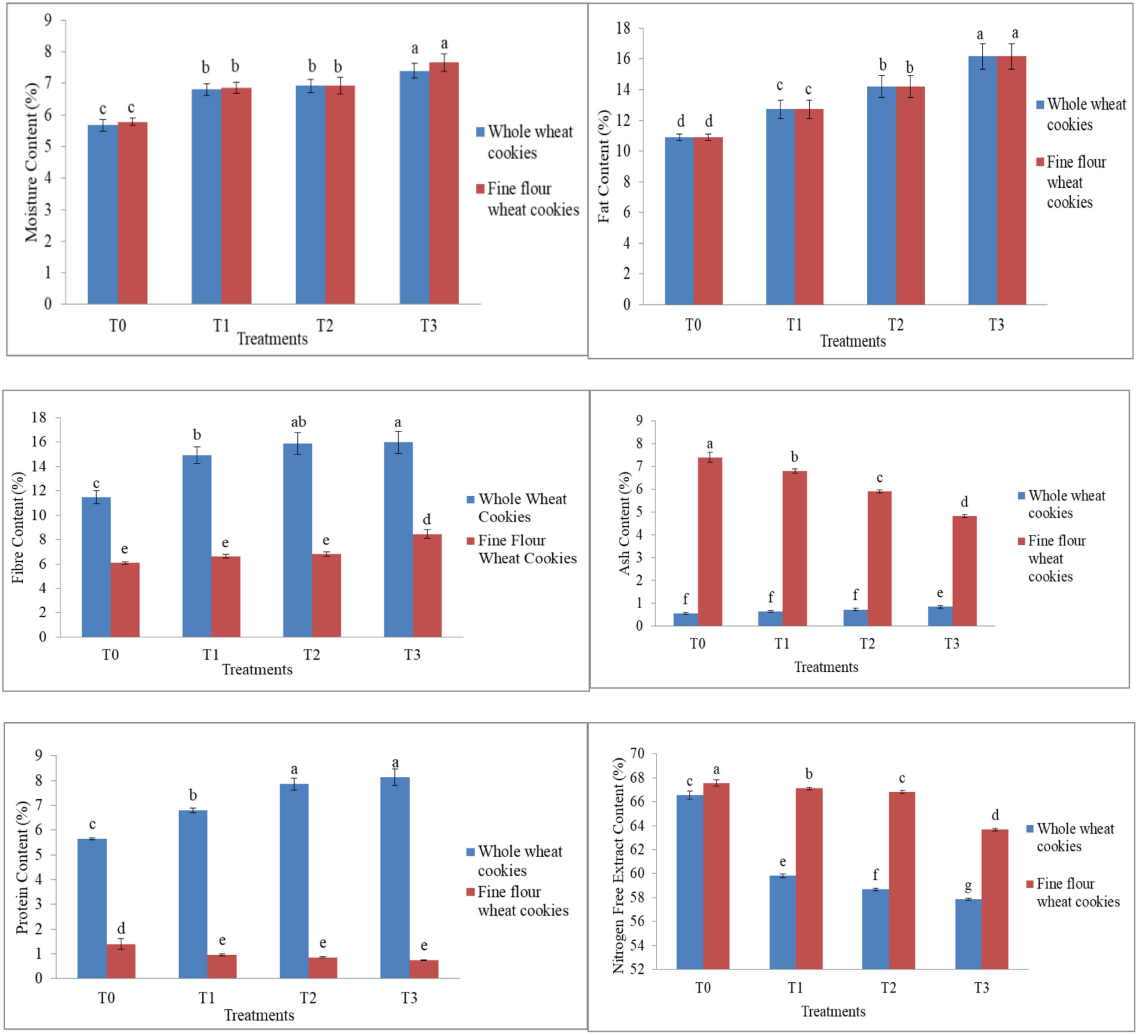
Comparative analysis between whole and fine wheat flour cookies for moisture contents, fat contents, fiber contents, ash contents, protein contents, and nitrogen extract (NFE) contents as affected by sesame levels. Lowercase letters (a, b, c, etc.) indicate statistically significant differences between treatments at the .05 significance level. Treatments sharing the same letter are not significantly different from each other, while treatments labeled with different letters are significantly different.

The moisture content of both cookies (whole/fine) increases gradually as the quantity of white sesame seeds increases and the level of wheat flour decreases. The statistical results showed that the results were nonsignificant (*p* > .05) for moisture in whole wheat and fine wheat cookies. Moisture content varies from 5.78% to 7.66% in fine wheat cookies, as shown in Figure 1. On the other side, the mean values of moisture content of whole wheat flour cookies with the same proportion of added sesame seeds were noted as 5.67%–7.39%. The moisture increases as the concentration of white sesame seeds increases because sesame seeds are good moisture absorbers and contain a good amount of moisture content. Our results were similar to those of Arshad et al. (2007), who reported that cookies made from wheat flour with defatted wheat germ flour on a replacement basis showed an increase in moisture content of 8.37%–9.02% with a nonsignificant difference between the treatments.

### 5.2 Fat content

The fat content was the same in fine wheat flour and whole wheat flour cookies. The mean value ranges between 10.89% and 16.16% in both whole wheat and fine wheat cookies, as shown in (Figure 1). No statistically significant changes in the fat analysis were found between cookies prepared with whole wheat flour and those made with fine wheat flour (*p* > .05). The mean values of the fat content of the whole wheat cookies showed a gradual increase as the amount of white sesame seeds was increased. This is because white sesame seeds are a rich source of oil, also called as the queen of oil seed crop. A similar increasing trend and results were found in fine wheat flour cookies with sesame seeds. T3 contains the highest value of fat content and T0 contains the lowest value of fat content. These results were consistent with the findings of Gernah and Anyam (2014), who reported a similar increasing fat content trend of 2.08%–5.23% in cookies made from wheat flour and sesame flour.

### 5.3 Fiber contents

The fiber content in fine wheat flour cookies is less as compared with whole wheat flour cookies. However, in both cases, the fiber content increased with the increase in the quantity of white sesame seeds. The statistical results indicated that the results are highly significant (*p* < .05) for the fiber contents of whole and fine wheat cookies (Figure 1). Significant increases in the total fiber contents of the resultant blends, from 11.47% to 15.98% in whole wheat cookies and from 6.1% to 8.46% in fine wheat cookies, are mostly attributable to the addition of sesame seeds in different proportions. These results are similar to those reported by Zouari et al. (2016) for cookies made from composite wheat and sesame seed peel flour, which showed an increase in the fiber content of cookies from 18.05% to 23.10% when using more sesame seeds in their formulation. Fiber content was increased because sesame seeds are a rich source of fiber content as compared to wheat flour.

### 5.4 Ash contents

The mean values range from 7.40% to 4.82% in fine wheat cookies, and the mean values of ash in whole wheat flour cookies range from 0.54% to 0.83%, as shown in Figure 1. The statistical results indicated that the results of the ash contents of whole wheat and fine wheat cookies were highly significant (*p* < .05). The ash level of fine wheat cookies ranges from the highest of 7.40% in the T0 treatment to a low of 4.82% in the T3 treatment. The highest ash contents in whole wheat cookies were in T3 (0.83%) and lowest in T0 (0.54%) because the majority of the grain's mineral content is found in the bran and germ, so whole wheat flour has higher ash concentrations. A similar trend was reported by Asim et al. (2018), who compared the ash content of several wheat types, including NARC‐2011, which showed a great amount of ash content. These findings are supported by Mutwali (2011), who reported that the ash content of flour decreased as the percentage of flour decreased in the formulation of cookies. Thus, fine wheat flour had more ash than whole wheat biscuits, but the addition of sesame seeds reduced the ash concentration.

### 5.5 Protein contents

The protein contents increase significantly in whole wheat flour cookies with increasing levels of sesame seeds but decrease in fine wheat flour cookies. The mean values of protein in whole wheat flour cookies range from 5.65% to 8.13%, and the mean values were between 1.40% and 0.74% in fine wheat cookies. Comparing the protein content of fine wheat cookies to whole wheat cookies, there is a statistically significant difference (*p* < .05). There is a possibility that high fiber protects the amino acids (protein) in whole wheat flour, causing an increased level of protein content in whole wheat cookies. There was a correlation between the amount of sesame seed replacement and the protein content. One possible explanation is the incorporation of a sesame seed, an oil seed that is also a rich source of protein. Sesame protein is well‐balanced in amino acids, with a chemical score of 62% and a net protein utilization of 54%. The decrease in the protein content of fine wheat flour cookies might be due to an increased level of the Maillard reaction between amino acids and reducing sugars. Another reason could be attributed to the low level of fiber in fine wheat cookies and high in whole wheat flour cookies. Thus, our findings are consistent with those published by Olagunju and Ifesan (2013), who reported an increased protein content of 15.18%–18.80% in cookies made from wheat flour and germinated sesame flour blends.

### 5.6 Total carbohydrate

The total carbohydrate decreases with an increased amount of sesame seeds in both fine wheat and whole wheat cookies. The mean values of NFE in fine wheat flour range between 67.55% and 63.67%. The mean values of NFE in whole wheat flour cookies ranged from 66.55% to 57.86%, as shown in Figure 1. Nitrogen‐free extract comparing whole‐wheat and fine‐wheat cookies showed statistically highly significant outcomes (*p* < .05). The average NFE in whole wheat cookies and fine wheat cookies (although with somewhat different means) both exhibited a reduction with increasing amounts of sesame seeds. White sesame seeds have been shown to hurt NFE, which may be due to the lower NFE contents in white sesame seeds as compared with wheat flour.

### 5.7 Phytochemical analysis of cookies

#### 5.7.1 Total phenolic analysis of cookies

**FIGURE 2:**
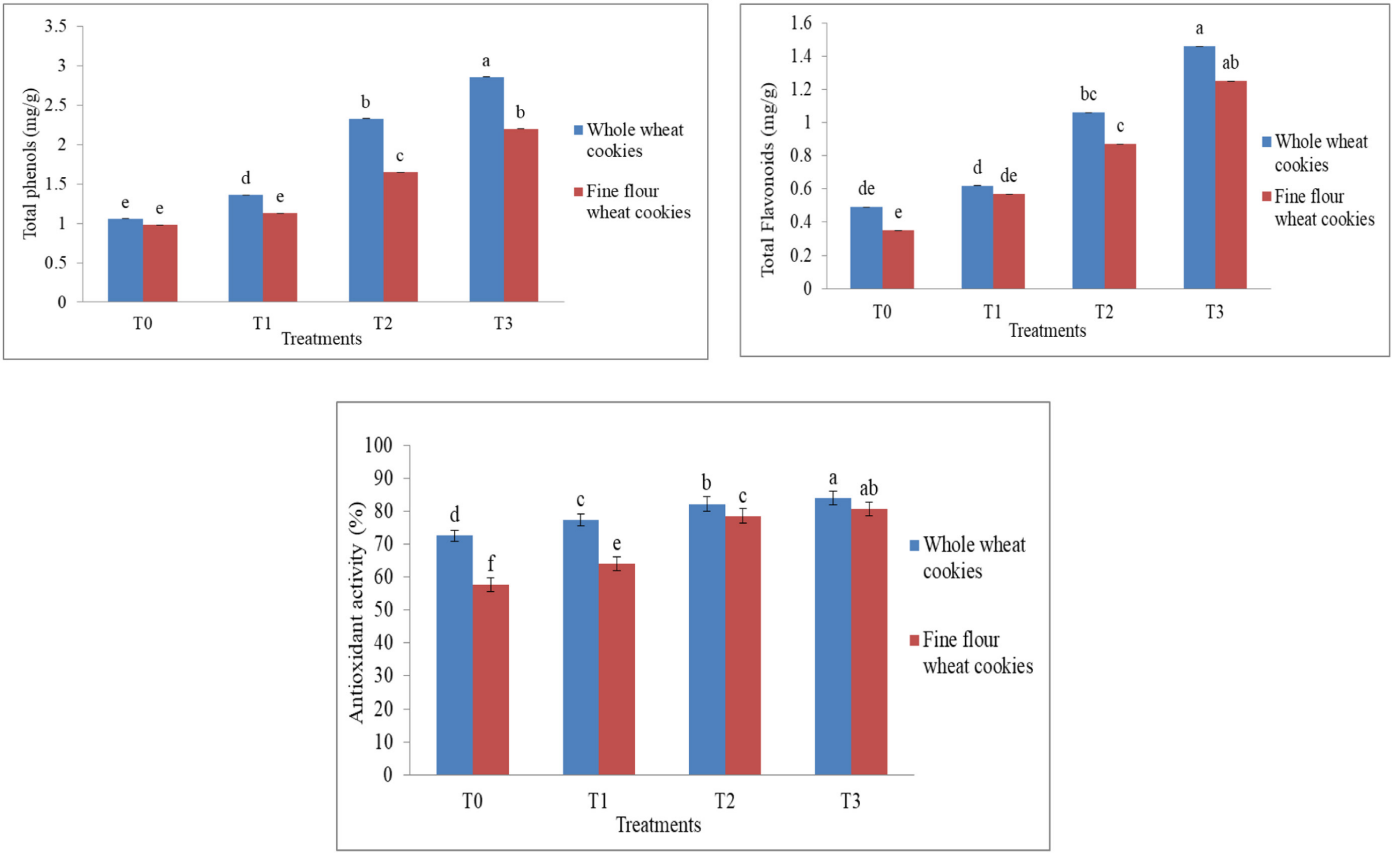
Comparative analysis between whole and fine wheat flour cookies for total phenolics, total flavonoids, and antioxidant activity as affected by sesame levels. Lowercase letters (a, b, c, etc.) indicate statistically significant differences between treatments at the 0.05 significance level. Treatments sharing the same letter are not significantly different from each other, while treatments labeled with different letters are significantly different.

The mean phenolic component levels in fine wheat flour and whole wheat flour cookies are shown in Figure 2. The statistical results showed that there was a highly significant difference (*p* < .05) between cookies made from fine and whole wheat flour. The phenolic content of cookies varied significantly when white sesame seeds were used at 5%, 10%, and 15%. In the whole wheat flour cookies formulation, the highest value was obtained in T3 (2.86%) and the lowest value was T0 (1.06%), while in the fine wheat flour cookies formulation, the highest value (2.20%) was obtained in T3 with 15% sesame seeds and the lowest value (0.98%) was in T0, which was the control treatment because mostly the phenolic contents are present in the husk and bran of wheat grains (López‐Perea et al., 2019). In fine wheat flour, the bran has been removed from the grain, but sesame seeds are a rich source of phenols, so the increased quantity of sesame seeds in fine cookies increases the phenolic content but in a lower percentage as compared with whole wheat cookies.

#### 5.7.2 Total flavonoid analysis of cookies

Statistical analysis showed a nonsignificant (*p* > .05) difference in flavonoids between whole wheat and fine flour wheat cookies, with variable results from T0 to T3. Flavonoids, many of which have anti‐oxidant qualities, are the subject of intense research because of their wide‐ranging positive effects on human health. The TFC value increases significantly because sesame seeds and whole wheat flour are rich sources of (phytochemical) flavonoids, so increasing the concentration of sesame seeds increases the flavonoid content in cookies. Total flavonoids in cookies made from wheat flour and sesame seeds followed a similar pattern reported by Agrahar‐Murugkar et al. (2018) and showed increased antioxidant activity and total flavonoids content compared to the control group 0.1 to 2.0 mg/g because sesame seeds are a good source of antioxidants (g‐tocopherol) phenols and flavonoids, which increase in the roasting process because of the formation of various low‐molecular‐weight phenolic and flavonoid compounds (Jeong et al., 2004).

#### 5.7.3 Antioxidant analysis of cookies

The mean values of antioxidant activity of cookies fortified with white sesame seeds present a highly significant difference (*p* < .05) for DPPH between all the cookies made from fine and whole wheat flour (Figure 2). 5%, 10%, and 15% of sesame seed utilization in cookies showed remarkable variation in their DPPH. The highest value of radical scavenging activity of DPPH (antioxidant activity) in whole wheat flour cookies was in T3 (84.09%) with 15% added sesame seeds. The lowest in T0 is 72.65%, having zero sesame seeds added because sesame seeds are rich in antioxidants (Elleuch et al., 2011), but the flour type also affects the flavonoid content in cookies differently. In fine wheat cookies, the highest value was noted in T3 (80.76%) with 15% sesame seeds, and the lowest value was 57.76% in control T0 with 0% sesame seeds. This result showed that white sesame seeds increased the antioxidant activity in cookies made from fine wheat flour due to the higher antioxidants in white sesame seeds. Lee (2020) also reported an increasing trend in antioxidants in cookies made from wheat flour and pak choi powder (8.23%–25.80%).

### 5.8 Fatty acid profile of wheat oil (NARC‐2011)

The fatty acid composition of whole wheat flour oil, wheat variety (NARC‐2011) is presented in Table 2. Oleic acid (C18:1) was found in the highest amount 45.76%, followed by linoleic acid (C18:2) at 37.46% and Palmitic acid (C16:0) at 16.76%. A sufficient percentage of unsaturated fatty acid was found in whole wheat oil.

**TABLE 2:** Fatty acids result from wheat oil (NARC‐2011) through GC.

S. No.	Retention time (min)	Carbon chain	Name of fatty acid	Percent peak area
1	14.476	16:0	Palmitic acid	16.76
2	17.43	18:1	Oleic acid	45.76
3	17.832	18:2	Linoleic acid	37.46
			Total	100

### 5.9 Fatty acid profile of sesame seed oil

The results of the fatty acid composition (as shown in the chromatograph of sesame seed oil shown in Figure 6 and Table 3) included linolenic acid (C18: 3) (an essential fatty acid belonging to the omega‐3 fatty acid group), which was the major fatty acid with the highest value 32.93% in white sesame seeds. The second highest fatty acids included oleic acid (C18:1), also called the monounsaturated omega‐9 fatty acids, with 28.302%. Linoleic acid (C18:2), also called the omega‐6 fatty acid polyunsaturated essential fatty acid, was found in 18.555%. Sesame seed oil also contained saturated fatty acids in reasonable amounts <10%. Palmitic acid (C16:0), arachidic acid (C20:0), and stearic acid (C18:0) were found in 10.501%, 4.852%, and 4.851%, respectively.

**TABLE 3:** Fatty acids result from sesame seed oil through GC.

S. No.	Retention time (min)	Carbon chain	Name of fatty acid	Percent peak area
1	14.565	16:0	Palmitic acid	10.509
2	17.433	18:0	Stearic acid	4.851
3	18.109	18:1	Oleic acid	28.302
4	18.268	18:2	Linoleic acid	18.555
5	18.386	18:3	Linolenic acid	32.93
6	18.826	20:0	Arachidic acid	4.852
	Total			100

### 5.10 Sensory analysis of cookies made from whole and fine wheat flour (NARC‐2011) fortified with white sesame seeds

Cookies made with 5%, 10%, and 15% of added white sesame seeds showed variations in their surface color. According to the findings, the sensory properties improved, but the value for color decreased as the quantity of sesame seeds increased. In fine wheat flour cookies, mean values range from 5.89 to 7.35. The color range of whole wheat cookies is 6.17–7.22. The browning effects of heat and enzymes caused the color value of whole wheat flour to decline when the number of sesame seeds was increased. Our findings are consistent with the research conducted by Hoojjat and Zabik (1984), who reported a decreasing trend of 6.30–5.8 in the color evaluation of cookies by the panelists as the level of sesame seeds increased and wheat flour decreased.

### 5.11 Taste

The taste scores of fine wheat cookies range from 6.63% to 8.56%. The flour type and environmental conditions also affect the taste of cookies. These results are similar to the findings of Hoojjat (1983), who reported the highest taste values in cookies with a 10% sesame seed level, as the panelists did not like the flavor of fine wheat cookies when more than 10% sesame was used in cookies. The flour type and environmental conditions also affect the taste of cookies. On the other side, the situation is different for whole wheat cookies; the mean values for taste range from 6.62% to 8.88%. The taste increases with the increase in white sesame seeds by up to 20%, enhancing the flavor/taste of cookies because the white sesame seeds are a little sweet with a nutty taste. This increased trend was related to the aftertaste of sesame‐fortified cookies liked by the panelists. These results are similar to the findings of Ndife et al. (2014), who reported a better taste in cookies with an increasing trend of 6.15–8.35, with the highest scores in T3 at 8.35 as the replacement of wheat flour increased in cookie formulation.

### 5.12 Texture

The texture analysis of fine wheat cookies was in the range of 6.63–8.82 cookies made from fine wheat flour. These results are similar to those of Nyadroh et al. (2021), who reported the same results of texture in cookies made from wheat flour, tiger nuts flour, and sesame seed, with a maximum score of 8.80 in treatment with 10% sesame seeds. In whole wheat flour, the texture analysis value ranges from 7.83 to 8.83. This result indicated that texture also increased with an increase in white sesame seeds in whole wheat cookies. Because sesame seeds add crunchiness and good texture to the cookies, increasing the sesame level in the cookies enhances the texture as well. Hafez (2018) also reported the highest texture value of 19.16 in treatment, with the highest level of sesame seeds at 20% in crackers made with wheat flour and defatted sesame flour, indicating that sesame seeds with increasing levels up to 20% showed the highest acceptability rate for texture.

### 5.13 Aroma

**FIGURE 3:**
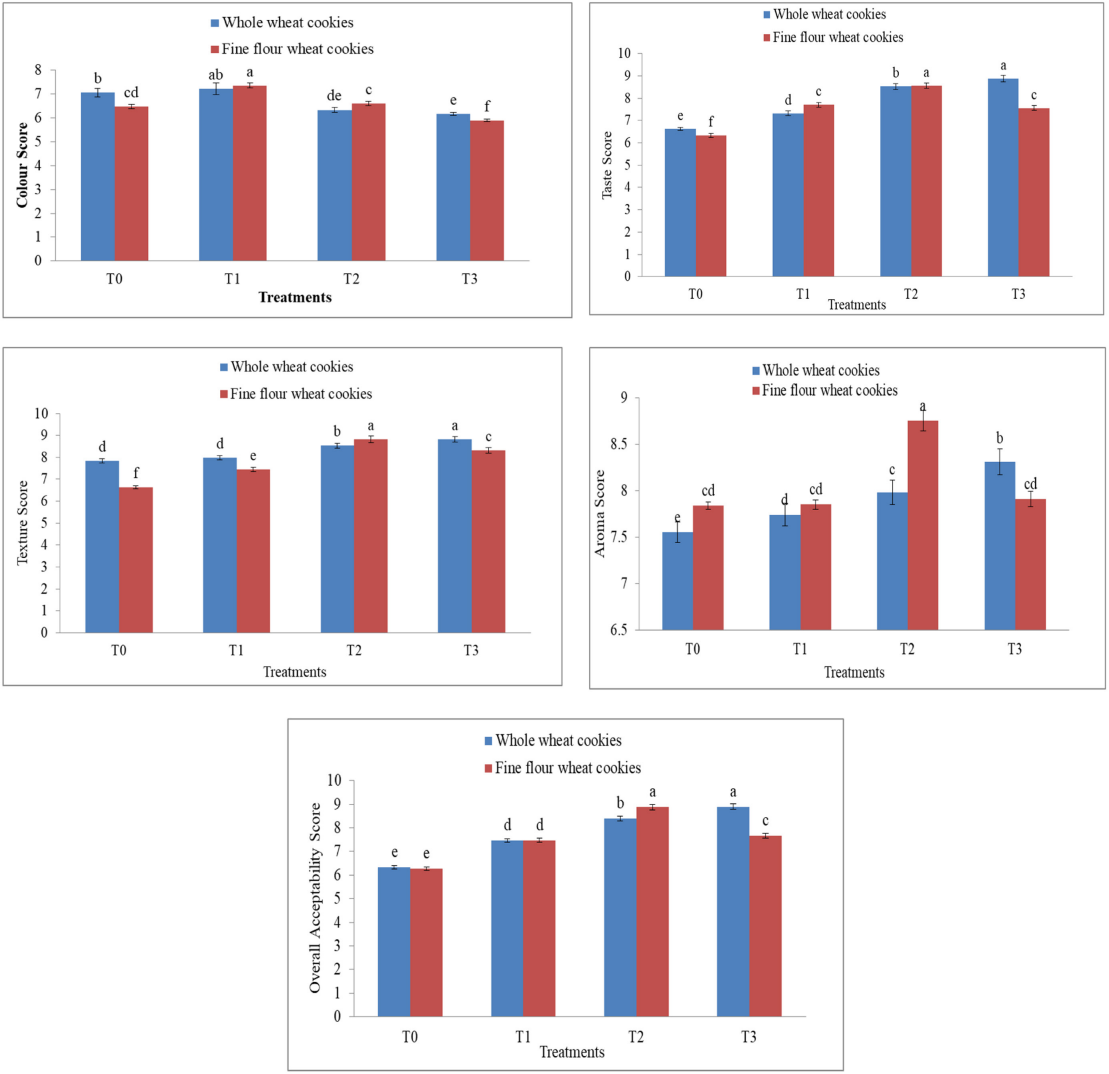
Differences in color, taste, texture, aroma score, and overall acceptability between cookies made from whole wheat flour and fine wheat flour cookies as affected by sesame level. Lowercase letters (a, b, c, etc.) indicate statistically significant differences between treatments at the 0.05 significance level. Treatments sharing the same letter are not significantly different from each other, while treatments labeled with different letters are significantly different.

In fine wheat cookies, the aroma ranges from 7.84 to 8.75. This means that increasing the sesame seeds has a positive effect on cookies. T2 was more acceptable than all the other treatments in fine wheat flour; this could be due to the enhanced flavor (nutty aroma) imparted by the sesame seeds. Akusu et al. (2019) showed a similar pattern of aroma in cookies composed of wheat flour and sesame seed flour. In whole wheat cookies, the aroma ranges from 7.55 to 8.31, as shown in Figure 3. The reason for the higher scores for the aroma and flavor of sesame seed was the presence of aromatic compounds such as tocopherols in it. Tocopherols are fat‐soluble compounds that promote the aroma and flavor of sesame seed cookies. The results were nearly similar to those of Ndife et al. (2014), who found the highest scores (8.33) in the cookies T3 treatment with maximum substitution of whole wheat flour and the lowest scores (6.25) in T0 (control treatment), which may be due to the nutty flavor and appealing aroma.

### 5.14 Overall acceptability

The analysis of the overall acceptability of fine wheat sesame‐fortified cookies ranged from 6.28 to 8.88. Whereas, in whole wheat cookies, acceptability varied from 6.33 to 8.89, with the maximum value acquired in T3 with 15% white sesame seeds and the lowest value received in T0 (Figure 3). According to the findings shown in Figure 3, T2 made from fine wheat flour and T3 made from whole wheat flour have the highest approval rates in terms of taste, texture, and aroma. It was determined that a higher percentage of sesame seeds improved the cookie's flavor, aroma, texture, and overall acceptability. The panelists rated the cookies containing sesame seeds less highly in terms of color because they believed the darker color came from overbaking. This was true of both fine wheat cookies and whole wheat cookies containing 15% sesame seeds. Rai et al. (2017) reported an increasing trend of 8.00–8.60 in overall acceptability with maximum scores in T3 treatment with the highest sesame seed level for cookies made from wheat flour, maize flour, and sesame seed.

### 5.15 Physical analysis of sesame‐fortified wheat cookies (whole flour/fine flour)

#### 5.15.1 Diameter

The statistical results were nonsignificant (*p* > .05) for the diameter between fine and whole wheat cookies. Physical attributes revealed that mean values for the diameter of sesame‐fortified whole wheat cookies were in the range of 48.47–52.31 mm. Cookies' composition is also strongly tied to their physical qualities. Cookies made with whole wheat flour had a wider diameter range than those made with fine wheat flour, but the diameter of cookies made with either kind of flour increased with the addition of sesame seeds. The diameter of fortified cookies in fine wheat flour cookies varies from 46.52 to 50.47 mm. These results indicated that the fat content of the sesame seeds or the decreased water absorption during mixing causes the cookie diameter to rise when using either fine or whole wheat flour to make cookies. These results are similar to the findings of Emam (2004) reported the same pattern in diameter as the level of sesame seeds was increased in cookies rises the diameter of cookies.

#### 5.15.2 Thickness

**FIGURE 4:**
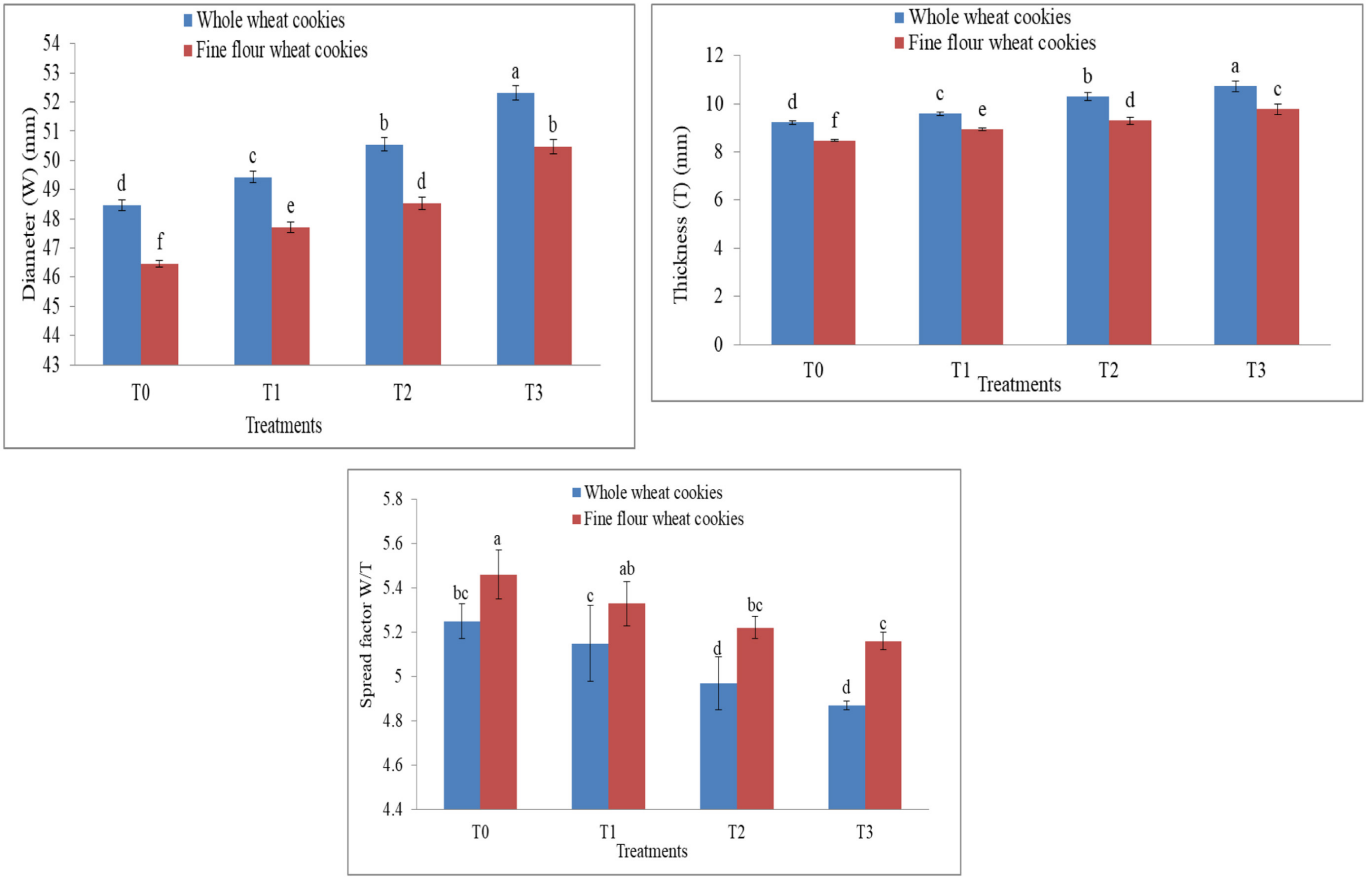
The difference in diameter, thickness, and spread factor between cookies made from whole wheat flour and fine wheat flour cookies as affected by sesame levels. Lowercase letters (a, b, c, etc.) indicate statistically significant differences between treatments at the 0.05 significance level. Treatments sharing the same letter are not significantly different from each other, while treatments labeled with different letters are significantly different.

The statistical result was significant (*p* < .05) for thickness in fine and whole wheat cookies. The mean values for whole wheat cookies for thickness indicated that T3 had the greatest value (10.73 mm) and T0, the control, had the lowest value (9.25 mm), as shown in Figure 4. Thickness values were highest in whole wheat flour cookies as compared with fine wheat cookies, but an increasing trend was noted for thickness in whole and fine wheat cookies. When the amount of sesame seeds was increased, the cookie thickness made from fine wheat flour rose from 8.45 to 9.77 mm. Cookies made with 30% sesame seeds had a thickness of 9.77 mm, whereas cookies made with T0 had a thickness of 8.45 mm. It is possible that the increased thickness of cookies is the consequence of water absorption, causing the flour components to expand and bind together. Prakash et al. (2018) reported an increasing trend of 7.70–9.98 mm for thickness as compared to the control treatment in cookies made from white sesame seeds and wheat flour due to an adequate expansion of the dough by leavening.

#### 5.15.3 Spread factor

**FIGURE 5:**
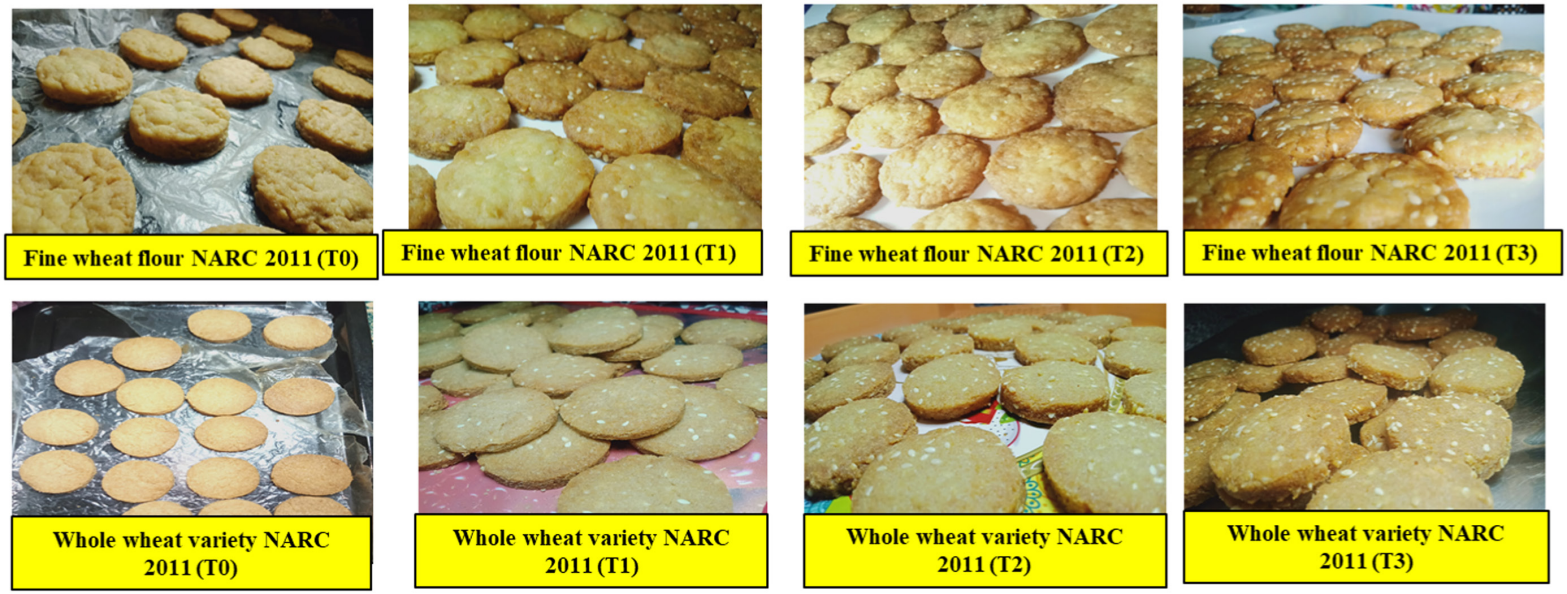
Pictorial presentation of cookies formulation (whole/fine wheat cookies).

**FIGURE 6:**
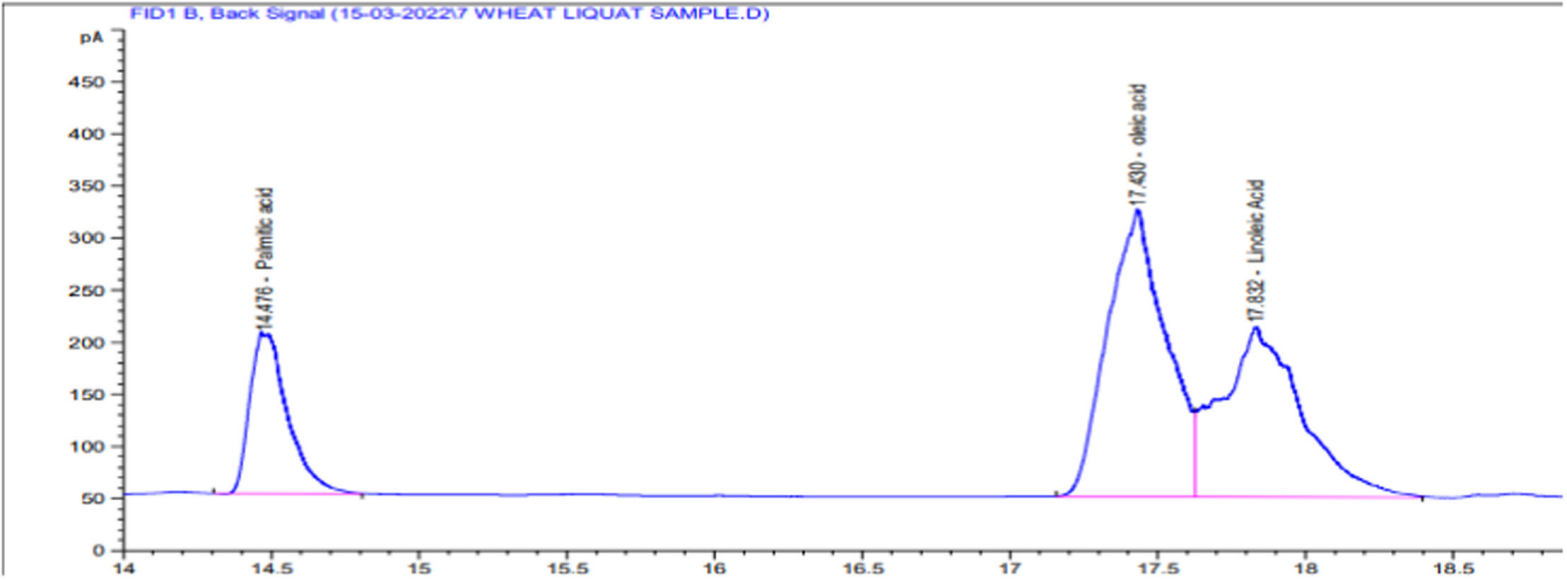
Gas chromatograph of wheat oil (NARC‐2011).

**FIGURE 7:**
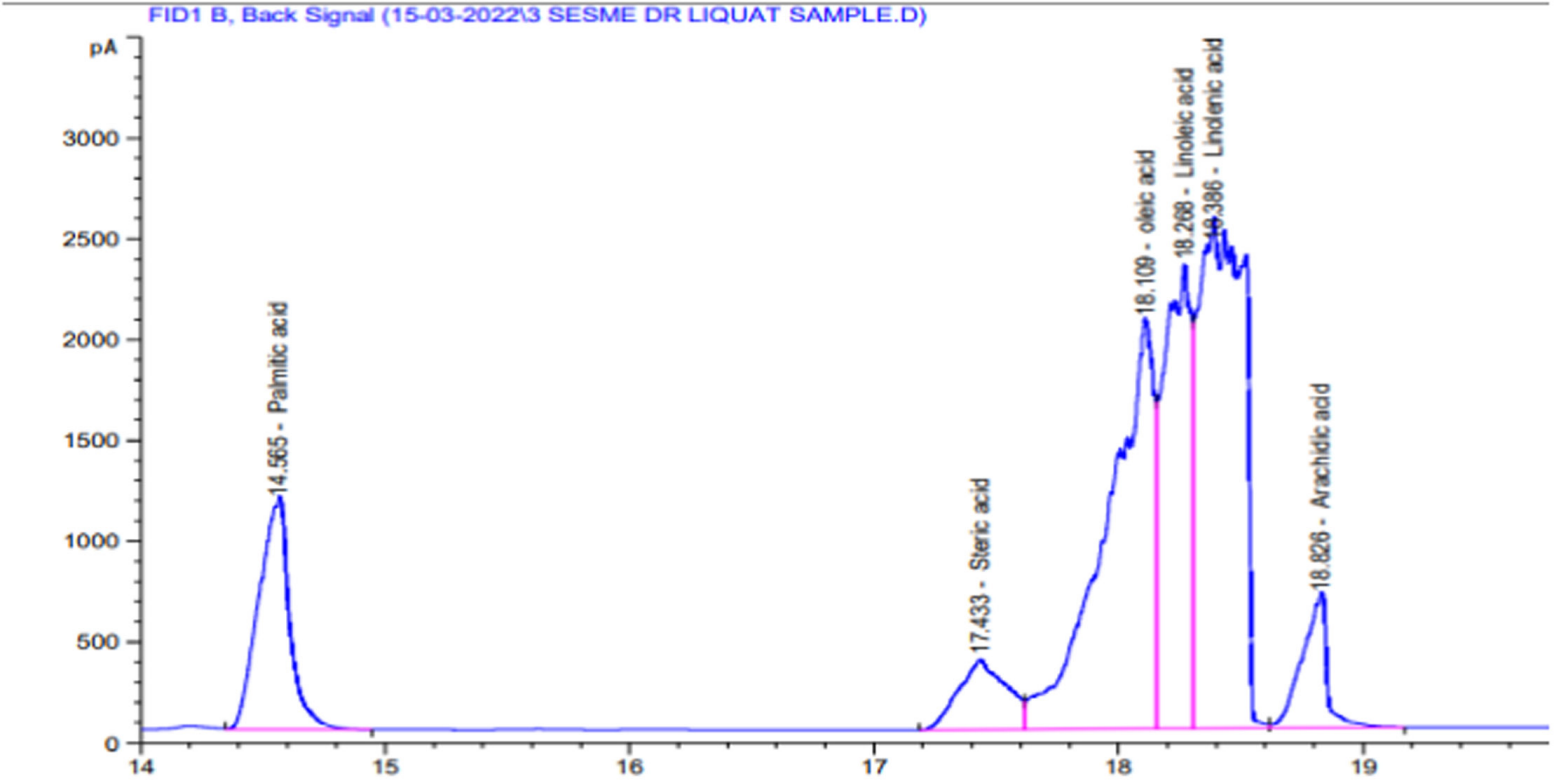
Gas chromatograph of sesame seed oil.

The statistical result was nonsignificant (*p* < .05) for the spread factor in fine and whole wheat cookies. The ratio that depends on the values of cookie thickness and cookie diameter is called the spread factor. The spread factor in whole wheat cookies ranges from 4.87 to 5.25. The spread factor for soft and tender fine wheat cookies ranges from 5.16 to 5.46. Fat and other functional properties may also affect spread. The high fiber and protein content in sesame seeds leads to a higher moisture absorption tendency of these biscuits, contributing to lower spread factors than the control biscuits. The spread factor of cookies with a decreasing trend of 46.7–37.3 mm was also reported by Kausar et al. (2018) with an increased replacement level of wheat flour with carrot pomace powder due to the higher fibrous content absorbing less water than that of plain wheat flour cookies, just like sesame seeds that have a great percentage of fiber and fat that causes the spread factor to decrease with the increased level of sesame seeds in cookies (Figures 5, 6, 7).

## 6 CONCLUSIONS

The present study demonstrated that among all the treatments and two types of flour in NARC‐2011 (fine wheat flour and whole wheat flour), the best treatment was selected, which was T2 in fine wheat flour and T3 in whole wheat flour and T2 with 10% of white sesame seeds and T3 with 15% of sesame seeds. These two treatments of fine and whole wheat flour are best for the production of cookies with satisfactory sensory attributes.

## AUTHOR CONTRIBUTIONS

**Sittara Noori Naqvi:** Data curation (lead); formal analysis (lead); investigation (lead); methodology (lead); software (lead); writing – original draft (lead). **Muhammad Liaquat:** Conceptualization (lead); data curation (equal); formal analysis (equal); investigation (equal); project administration (lead); supervision (lead). **Abeer Kazmi:** Conceptualization (lead); data curation (lead); formal analysis (lead); visualization (lead); writing – original draft (lead). **Shella Sammi:** Data curation (equal); formal analysis (equal); investigation (equal); methodology (equal); writing – original draft (equal). **Amir Ali:** Conceptualization (equal); data curation (equal); project administration (equal); visualization (equal); writing – original draft (equal); writing – review and editing (equal). **Juan Pedro Luna‐Arias:** Conceptualization (lead); investigation (equal); project administration (equal); supervision (lead); validation (equal); visualization (equal); writing – review and editing (lead). **Izzat Ullah Sherzad:** Conceptualization (equal); project administration (equal); software (equal); supervision (equal); writing – original draft (equal); writing – review and editing (lead).

## CONFLICT OF INTEREST STATEMENT

The authors declare that they have no known competing financial interests or personal relationships that could have appeared to influence the work reported in this paper.

## ETHICAL APPROVAL

In the sensory evaluations conducted for the cookies produced in this study, we rigorously followed established ethical guidelines that have been approved by the ethical committee of the University of Haripur. We affirm that our study fully adheres to all pertinent regulations, and prior informed consent was diligently obtained from all participants involved in the collection of these samples.

## Data Availability

Data will be made available on request.
